# A single-center experience with linear accelerator-based stereotactic radiotherapy for meningiomas: hypofractionation and radiosurgery

**DOI:** 10.1007/s00432-022-04450-y

**Published:** 2022-10-28

**Authors:** Ahmed Gawish, Nurlan Abdulayev, Souhir El-Arayed, Burkard Röllich, Hans-Joachim Ochel, Thomas B. Brunner

**Affiliations:** 1 Department of Radiation Oncology, University Hospital Magdeburg, Leipziger Str. 44, 39120 Magdeburg, DE Germany; 2 Department of Radiation Oncology, Medical University of Graz, 8036 Graz, Austria

**Keywords:** SRS, Radiosurgery, Hypofractionated radiotherapy, Meningioma

## Abstract

**Purpose:**

Meningioma is a common type of benign tumor that can be managed in several ways, ranging from close observation, surgical resection, and various types of radiation. We present here results from a 10 year experience treating meningiomas with a hypofractionated approach.

**Materials and methods:**

To define the rate of tumor control and factors associated with the relief of symptoms and radiation-related complications after radiosurgery and hypofractionated radiosurgery for patients with imaging-defined intracranial meningiomas. We reviewed the charts of 48 patients treated with stereotactic radiosurgery (SRS) or hypofractionated stereotactic radiotherapy (SRT) from 2002 to 2018. A total of 37 (82%) patients had WHO Grade 1 disease, and 11 (22%) had Grade 2. Outcomes that were analyzed included local control rates and the rate and grade of any reported toxicity.

**Results:**

Only 36 patients with 38 lesions, who underwent the follow-up regime, were enrolled in the retrospective analysis. The follow-up mean was 40 months (12–120 months). 25/34 patients had surgery before the radiotherapy. Sixteen underwent SRS with a median dose of 13, 5, and 20 received hypofractionated SBRT with a median dose of 26.9 (22-45 Gy) in median six fractions (5–13 fractions). Local control at 2 and 5 years for all patients was 90 and 70%, respectively. No patient suffered from toxicity > 2 CTC. 21/36 patients showed stable disease, while 8/36 patients showed partial Remission. 7/36 developed recurrent meningioma (five in-field), only one patient with grade 1 meningioma, in a median of 22 months (13–48 months).

**Conclusion:**

SFRT was superior to SRS for local control in our analysis of Grade I meningiomas. This might be due to a tendency for higher EQD2 in the PTV with SFRT compared to SRS, which was reduced to avoid brain necrosis in large PTVs. Therefore, SFRT appears preferable for typical meningioma PTVs.

## Introduction

Meningioma is the most common primary central nervous system tumor accounting for about 37.6% of them and approximately 50% of all benign brain tumors (Ostrom et al. 2019). They are classified into three grades according to the World Health Organization (WHO). They are mostly benign, with the majority (82–92%) classified as Grade 1. About 1–3% of meningiomas can turn malignant with a 5 year survival rate between 32 and 64% (Ostrom et al. 2019). However, their benign nature, and their position in the central nervous system can cause serious problems that require medical Treatment. It is critical to strike the right combination between therapeutic intervention and minimizing therapeutic-related neurologic damage while designing the therapy plan for meningioma patients (Ostrom et al. 2019). As a consequence, small, asymptomatic meningiomas are best left untreated, with the treatment reserved for clinical or radiological progress. Surgery, surgery with postoperative radiation therapy, or stand-alone radiation therapy may be used to treat patients who require immediate treatment (Wiemels et al. 2010; Asayel 2021; Lim et al. 2013). In expressions of radiotherapy, stereotactic radiosurgery (SRS) is a therapeutic option that provides a great conformal dosage of radiation in a sole portion by the speedy decline, preserving the healthy brain parenchyma or important adjoining structures (Lim et al. 2013). As a result, SRS has a long history of use in the treatment of meningiomas (Leksell 1951; Kondziolka et al. 2018; Korah et al. 2010; Pollock et al. 2013). When there is a risk of healthy nerve damage as a result of a big tumor mass or anatomic proximity to vital brain areas, standard fragmented radiation is often used. Hypofractionated stereotactic radiation (SRT) has lately emerged as an additional therapeutic option. SRT, which is a fractionated version of SRS, enables conformal, great dosage per portion radiation to be delivered in three to five portions (Lo et al. 2020; Lee et al. 2002; Milker-Zabel et al. 2005; Bria et al. 2011). When compared to SRS, the available data on this method are minimal; nevertheless, the consequences seem to be encouraging in terms of safety with minimal side effects as well as effectiveness. The main difference between the GK and linear accelerator-based SRS/SRT, that the GK technique uses similar marginal doses but with a significantly higher maximum point dose given a prescription to the 50–65% isodose line, while the linear accelerator-based SRS has a more homogeneous isodose and prescription coverage (Park et al. 2013; Han et al. 2017).

Patients diagnosed with meningioma and treated with radiation at our institution have frequently been treated with this SRT technique throughout the last decade. The findings of our experience with SRT in the treatment of meningioma are shown below.

## Materials and methods

For planning the treatment, the following procedures were performed. A contrast enhancement with gadolinium, T1-weighted neuro-navigation magnetic resonance imaging (MRI) with a thickness of 0.5 by 0.5 mm and a width of 1 mm was done before treatment. A planning computer tomography (CT) simulation was done after the MRI examination. Patients were immobilized throughout the planning CT simulation using a Brainlab (Feldkirchen, Germany) repositioning mask technology. To minimize head tilt motion, a mouth bite apparatus was inserted against the top teeth. If the patient could not bear the existence of the mouth bite, it was not used. Furthermore, a personalized thermoplastic mask was created to fit the patient's head features. CT images were taken once the subject was immobilized, with a resolution of 1 mm by 1 mm and a slice thickness of 2 mm. The images were co-registered to the MRI data set in the Brainlab iPlan visualization software after the capture of CT images. After that, the radiation oncologist drew the gross target volume (GTV), which was then extended by 2–5 mm margin to produce the preparation target volume (PTV).

### Treatments

Elderly or weak patients judged refractory of conventionally fractionated radiotherapy over 6 weeks and unsuitable for the operation were provided hypofractionated radiotherapy or SRS. All patients received photon external beam treatment. CT and MR registration were used to imitate patients. The gross tumor volume encompassed the total volume of all viable tumors. A clinical volume goal of 0–5 mm was used (0 mm for SRS, 5 mm for HFRT). With daily cone beam CT imaging guidance, a planned target volume of a 2–5 mm was employed.

### Statistical analysis

The study database was closed for analysis on January 1, 2022. Treatment toxicity (radiologic or symptomatic edema), progression-free survival (PFS), and overall survival were the study’s objectives (OS). The baseline attributes and frequency distributions were provided in a descriptive manner. The Wilcoxon rank sum test was used to compare continuous data. By inverting the censoring variable, the median follow-up was calculated. PFS and OS were estimated using the Kaplan–Meier technique starting on the first day of RT. The log-rank test was used to make survival comparisons. After stratifying by tumor grade, the cumulative incidence of local failure (LF) was assessed; fatalities without documented disease progression were considered as a competing event. Gray’s test was used to compare LF. Patients who are still alive or who have been lost to follow-up were censored. SAS version 9.4 was used to conduct the statistical analysis (Cary, NC, USA). Equivalent dose in 2-Gy fractions (EQD2) calculations were performed using the linear–quadratic equation and the RBApp application (Lee et al. 2002).

## Results

We identified 45 patients with 48 meningiomas who had SRS or SFRT between the years 2002 and 2018 in our center. Only 38 lesions (36 Patients) had an adequate follow-up, so we excluded the rest of the patients. A total of 11 patients (27%) had a confirmed grade 2 meningioma, whereas five patients had pathologically proven grade 1 meningioma, and the remainder had radiologically presumed grade 1 meningioma (Table1).

**Table 1:** Patient characteristics

Patient characteristics, *n* = 36	Value
Male	17
Female	19
Planning target volume (cc)	Median 6 (2, 3–22, 6)
Age	41–84
WHO grade 1	26
WHO grade 2	10

Most patients (20/36) were treated with a hypofractionated approach (SRT) due to their old age and request for a shorter treatment regimen with a median dose of 26.9 Gy with a median of 5 (mean six fractions; range: 22–39 Gy in 5–13 fractions). The median equivalent dose in 2 Gy fractions with an assumed alpha/beta ratio of 2 Gy was 58.31 Gy (range: 48.75–110 Gy) for SRS and 43.75 Gy (range: 42.24–48.75 Gy) for hypofractionated radiotherapy. Sixteen patients were treated with SRS (single fraction) median dose was 14 (13–20) Gy. In terms of tumor size, the median planning tumor volume was 6 ml (2.3–22.6) ml. The median follow-up after completion of radiation treatment was 41 (12–120) months. Every patient had follow-up MRI imaging available for review, with a median number of available MRI scans being 5 (1–16).

Fifteen patients had previously undergone surgery. Dexamethasone therapy was used prior to and during RT. Dexamethasone was increased in six patients during RT whose having had anti-edematic-therapy therapy with dexamethasone from the start of RT to manage worsened neurologic signs of edema. Thirteen individuals reported unchanged or better symptoms following radiotherapy. Treatment was well tolerated with no grade ≥ 3 acute toxicity and no evidence of late Grade ≥ 3 toxicity in the form of radio necrosis.

### Outcome

**Fig. 1:**
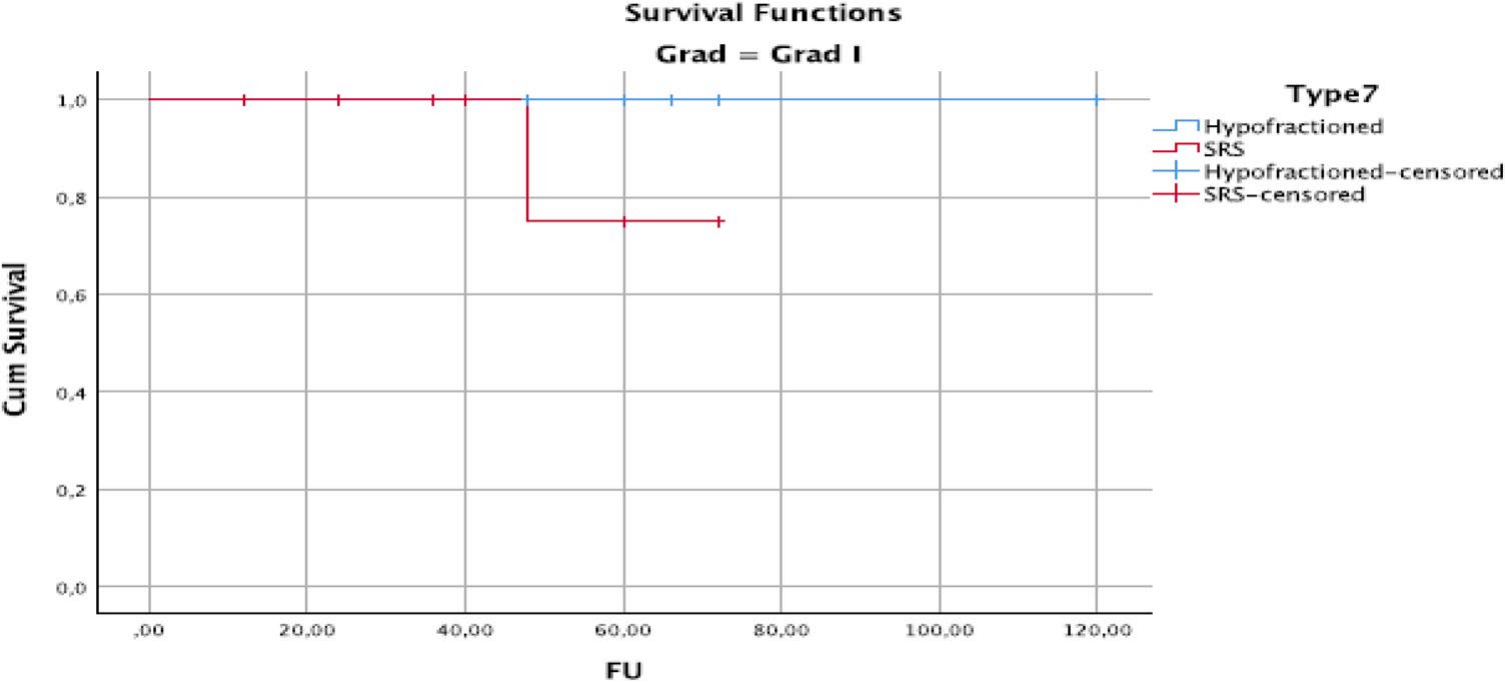
Kaplan–Meier plot of overall survival for grade I meningioma by fractionation: stereotactic radiosurgery (SRS) versus non-SRS

Local control for the whole group was 100% at 12 months, 95% at 24 months, and 70% at 5 years (Fig. 1). Seven people developed recurrent meningioma (five in-field), one patient with grade 1 meningioma, after 48 months, and six patients with Grade 2 meningioma had a local failure, after a median of 36 months (range 12–60 months). Five underwent re-resection, and two underwent fractionated radiation. All seven patients were alive at the end of follow-up; however, 8/36 of the remaining patients died without radiographic progression from intercurrent reasons of death.

**Fig. 2:**
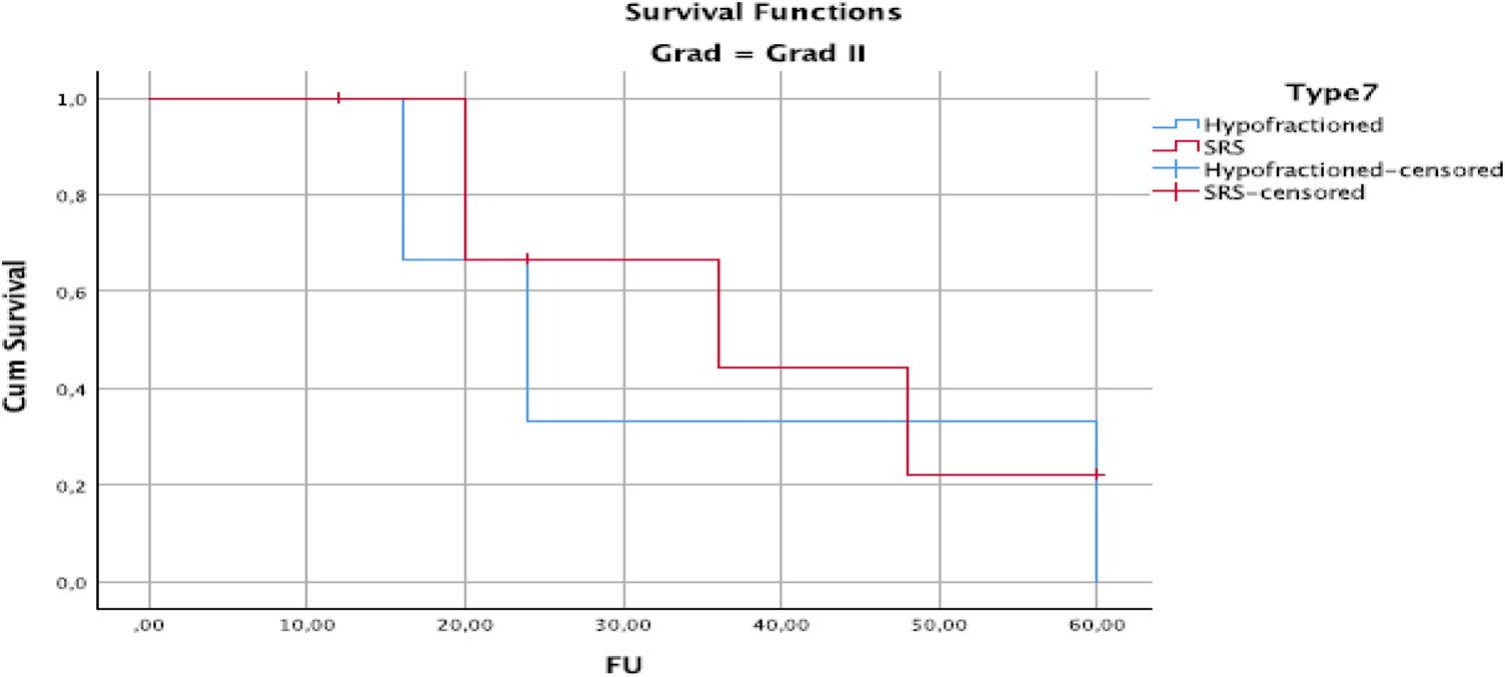
Kaplan–Meier plot of overall survival for meningioma grade II meningioma by fractionation: stereotactic radiosurgery (SRS) versus non-SRS

**Fig. 3:**
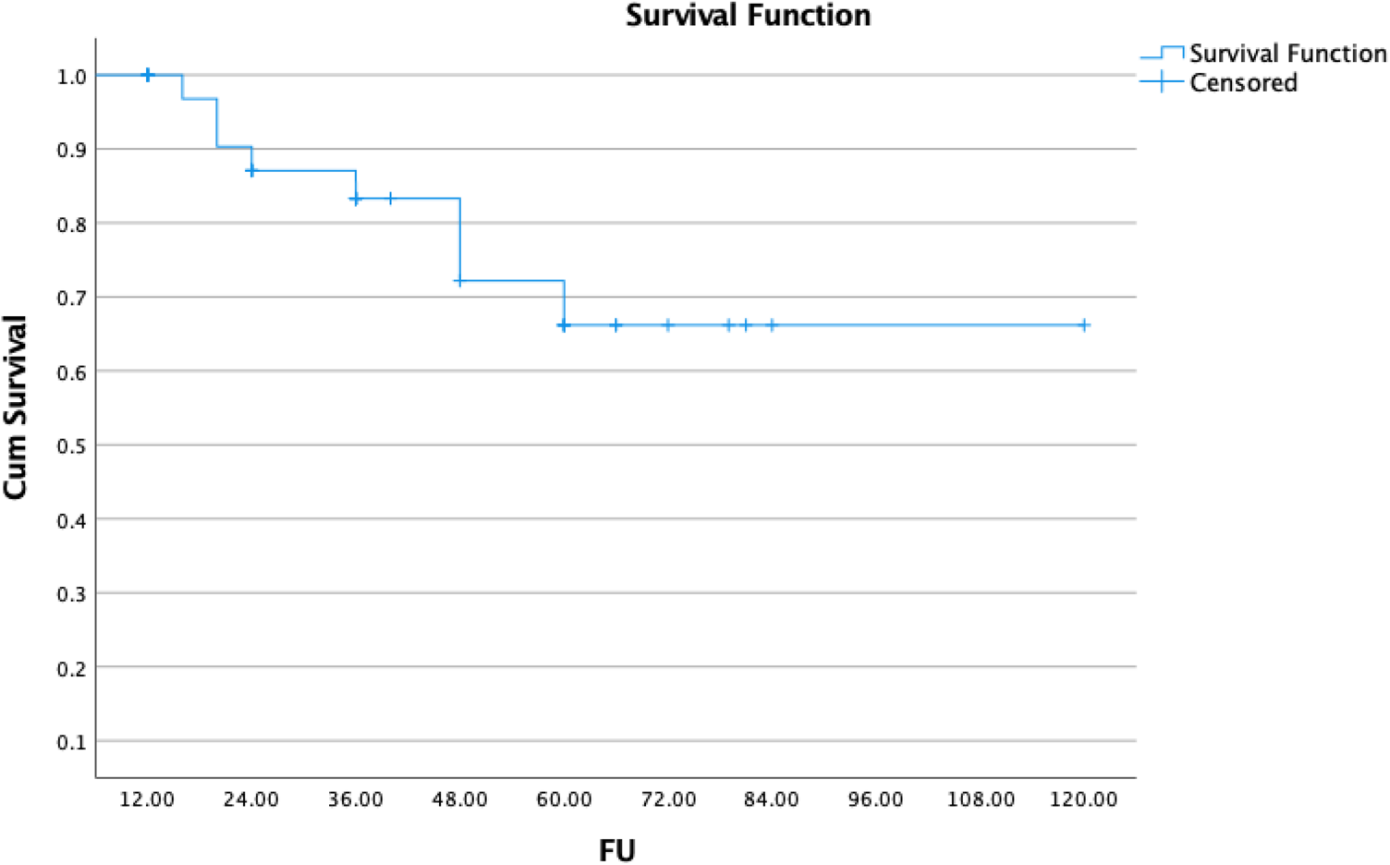
Kaplan–Meier plot of overall survival for meningioma by fractionation: stereotactic radiosurgery (SRS) versus non-SRS

At univariate analysis, HFRT treatment versus SRS led to improved local control, with a local control rate of 90% compared to 50% at 5 years, *P* = 0.02 (Fig. 2). Meningioma grade 2 had poor local control after both SRT and SRS, with a controlled rate of 35 and 22% after 60 months of follow-up (Fig. 3).

Multivariate analysis confirmed fractionation as a predictor for improved local control, hazard ratio (HR) = 0.003, 95% confidence interval (CI), and *P* = 0.007. At multivariate analysis (Table 2), predictors of local failure were lesions with grade 2 and patients without prior resection (*P* < 0.05). Target volume size, age at the time of SRS, and sex were not predictive of local control. Likewise, no predictors of increased toxicity were identified.

**Table 2:** Multivariate analysis and prediction of local failure

Factor	*P* value	95% confidence interval
Age (years)	0.45	0.959–1.024
Fractionation (HFRT vs SRS)	0.007	0.982–1.070
Grade (I/II)	0.003	0.957–0.997
Operation before SRS	0.05	1.076–1.123
Volume (PTV) (ml)	0.045	0.975–0.999

## Discussion

Our analysis shows better local control with SRT compared to SRS four grade 1 meningioma. This is an interesting finding: our radiation dose analysis reveals that patients who were treated with SRS received a lower equivalent radiation dose (EQD2). This is most probably a consequence of a dose compromise to avoid brain necrosis after SRS. Despite the location of meningiomas on the surface of the brain, the PTV volumes are often large and thus involve significant volumes of normal brain tissue. We conclude that SRT hopefully superior to SRS for the treatment of low-grade meningioma. Furthermore, grade 2 meningioma appears to require higher doses of radiation as reflected by the significant poorer local control rate in our cohort.

Meningiomas are the most common central nervous system tumors, and most of them are WHO Grade I benign tumors. Meningioma is frequently incidentally discovered after a cranial imaging was performed for another reason, i.e., after trauma. Even though most meningiomas are benign, they might show symptoms if they cause mass effects or are located adjacent to vital areas such as the brainstem or the optic pathway. If the size is small and in a good anatomic position, watchful waiting is usually the best course of action (Ostrom et al. 2019; Wiemels et al. 2010; Asayel 2021; Lim et al. 2013).

A study from Germany monitored approximately 60 patients with meningiomas that were identified accidentally and found that they grew roughly 2 cm^3^ per year. It was noticed that the early tumor size was not an indicator of the rate of growth, implying that non-surgical treatment with follow-up is a viable option. When the follow-up MRI imaging shows signs of increasing in size, surgery is mostly the preferable approach, if the Tumor is at an operable region (Herscovici et al. 2004; Nakamura et al. 2003). While performing the surgery, the extension of resection should be evaluated using Simpson evaluation. The extent of resection has a big influence on the rate of recurrence (Nanda et al. 2016). Grade 3 and 4 has higher recurrence rate that can be as high as 30–35%. In such cases, close follow-up is required, with adjuvant therapy in the form of radiotherapy being considered (Rogers et al. 2015). A study published from Mayo Clinic with 570 patients treated surgically demonstrated a 75% progression-free survival rate after 10 years, with a 2% perioperative death rate (Stafford et al. 1978). Another study published from Finland looked at the results of over 900 individuals who had intracranial meningiomas surgically removed and found that 90% of them had outstanding long-term local control, while under 7% had a surgical mortality (Kallio et al. 1992).

Radiotherapy can also be performed without surgery to treat meningioma as a first line of treatment for the selected cases, with a more traditional technique of 50 Gy delivered in 25–28 daily portions (Kondziolka et al. 2018; Korah et al. 2010). At a median continuation of 5.7 years, local control rates in a collection of over 400 patients preserved with a median dose of 58.6 Gy were 93%. About 40% of individuals with underlying neurologic deficits improved after treatment, and 10% of the patients suffered from symptom aggravation (Leksell 1951; Kondziolka et al. 2018; Korah et al. 2010; Pollock et al. 2013).

With the advancement over the years, the treatment with gamma knife then Cyberknife and linear accelerator-based technologies has increased and is very important to be considered while planning the therapeutic options for the patients. Many studies were performed to review the outcome after treating the meningiomas with Gamma knife stereotactic SRS.

Using a single fraction with a median dose of 15 Gy for treatment of 100 patients with larger volume meningiomas (defined in this study > 10 cm^3^) proved a local control of 92% after 7 years, reported by Bledsoe et al. (2010). Unfortunately, 23% of patients suffered complications like hemiparesis, cranial nerve damage, cerebral infarction, and hearing loss. Therefore, the treatment SRS for such large lesions was not recommended, and primary surgical removal should be the primary line of treatment [Bledsoe et al. 2010].

The same group of authors published a study from the Mayo Clinic. A total of 600 patients with 80% having proved or suspected Grade 1 meningiomas were treated over the course of 18 years. The average dose given was 16 Gy. About 11% of the patients developed radiation-related side effects. Otherwise, the therapy was well tolerated, and local control was achieved for 94% of the patients (Pollock et al. 2013).

Another study from the University of Pittsburgh reviewed about 900 patients with over 1000 meningiomas, the majority with grade 1 meningiomas, who were treated using SRS for nearly 20 years (Park et al. 2013). Local control was achieved by about 93% of the patients after 18 years and the therapy was good tolerated with just 4% of patients experiencing radiation-related complications (Kondziolka et al. 2018).

With the development in radiotherapy, hypofractionated stereotactic radiotherapy (SFRT) strategy has been gradually incorporated into the management of brain lesions. Many authors describe it as a safer technique, especially in big tumors in high-risk areas.

The Gamma knife was generally used for single-fraction therapy. Nowadays, it is increasingly widely used in hypofractionated therapy (Park et al. 2013; Han et al. 2017). A group from Seoul, South Korea, published the results after the treatment of 23 patients with meningiomas larger than 10 cm^3^ using hypofractionated a gamma knife. The median dose was 19 Gy in three portions, with a 38 month follow-up. During follow-up, no patients reported local failure. About 17% of the patients experienced temporary cerebral neuropathy (mostly trigeminal) as a result of the treatment (Park et al. 2013).

Similarly, another South Korean group reported the results of 70 patients with tumors larger than 10 cm^3^ using gamma knife. Sixty percent of the patients were treated in a single fraction to a dose of 12 Gy, while the rest were administered in two-to-five fractions with total doses varying from 16 to 18 Gy. At 5 years, the fractionated group had a mathematically greater percentage of local control than the unfractionated group (93 percent versus 88%, *P* = 0.389) with less complication rate of 8–34% (Bria et al. 2011).

One of the most recent studies from the University of Pittsburg reviewed 56 patients after treatment using SRS or SRT. It showed evidence of increased local control of 88% after 5 years with minimal toxicity through the use of hypofractionated radiotherapy (Wegner et al. 2019).

Another study from Pittsburgh looked at the results of 74 meningioma patients with 82% grade 1 meningioma who were treated with a median dose of 24 Gy in three fractions using the Cyberknife. At 1 year, local control for grade 1 lesions was about 96%, with just one incidence of late-grade three toxicity (Bria et al. 2011).

Researchers from Germany and Italy remarked on a big group of patients who were treated hypofractionated and had good rates of local control (more than 90%) and low cytotoxicity (6%) (Maranzano et al. 2015; Fokas et al. 2014).

A current trial, with a median follow-up of 64.7 months, involved 62 patients (aged 26–87) with 67 tumors who were treated with the CyberKnife and had a planned treatment course of 3–5 fractions with 18 to 25 Gy (Nguyen et al. 2021). The 5 year LC and OS were, respectively, 85.2 and 91.0%. The rates of radionecrosis and late-grade III/IV toxicity were 3.2 and 4.8%, respectively. In the most recent Italian study, 178 patients were enrolled prospectively, and underwent HSRT, which was with schedule of 25 Gy in 5 fractions. A median follow-up is to 53 months (range, 4–101 months) (Pinzi et al. 2022). Overall, there was a 12.7% rate of toxicity (21 of 166 patients), the 5 years local control rate was 97% (95% confidence interval, 92–99%).

Another study from Italy looked at the results of 52 patients who were treated using single-fraction SRS for lesions smaller than 2 cm and fractionated therapy for lesions larger than 2 cm. Local control was achieved for 92% after 4 years. There was no difference between single-fraction and hypofractionated regimens (Franco et al. 2018).

The results of our study are similar to the results mentioned above and strengthen the collected data. Grade 1 meningiomas treated with hypo-fractioned radiotherapy and SRS has shown a local control for the patients of 100% at 24 months and 75% in 5 years. The treatment was well tolerated with no Grade 3 acute or late toxicity. On the other hand, using hypo-fractioned radiotherapy and radiosurgery for grade 2 meningiomas showed local failure (Table 3).

**Table 3:** Different radiation regimes and EQD2

Radiation regime	Alpha/Beta 2	Alpha/Beta 3	Alpha/Beta 10
13 × 3 Gy	48.75	46.8	46.8
6 × 4, 4 Gy	42.24	39.07	31.68
5 × 5 Gy	43.75	40	31.25
1 × 13 Gy	48.75	41.6	24.92
1 × 13, 5	52.31	44.55	26.44
1 × 15 Gy	63.75	54	31.25
1 × 20 Gy	110	92	50

## Conclusions

This work adds to the growing body of evidence for high rates of local control and low toxicity when adopting an SRS or hypofractionated stereotactic radiotherapy for the treatment of low grade especially WHO grade 1 meningiomas. Our data shows high percentage of local failure after using SRT for treatment of WHO grade 2 meningiomas.

## Data Availability

The datasets used and analyzed during the current study are available from the corresponding author on reasonable request.

## Abbreviations

WHO: World health organization
SRS: Stereotactic radiosurgery
OS: Overall survival
LF: Local failure
PFS: Progression-free survival
EQD2: Equivalent dose in 2-Gy
SRT: Hypofractionated stereotactic radiotherapy
GK: Gamma knife
PTV: Planning tumor volume
RT: Radiotherapy
